# Recent advancements and future requirements in vascularization of cortical organoids

**DOI:** 10.3389/fbioe.2022.1048731

**Published:** 2022-11-03

**Authors:** Erin LaMontagne, Alysson R. Muotri, Adam J. Engler

**Affiliations:** ^1^ Department of Bioengineering, University of California, San Diego, La Jolla, CA, United States; ^2^ Department of Pediatrics, University of California, San Diego, La Jolla, CA, United States; ^3^ Department of Cellular and Molecular Medicine, University of California, San Diego, La Jolla, CA, United States; ^4^ Sanford Consortium for Regenerative Medicine, La Jolla, CA, United States

**Keywords:** brain organoid, vasculature, tissue engineering, organ-on-a-chip, microenvironment

## Abstract

The fields of tissue engineering and disease modeling have become increasingly cognizant of the need to create complex and mature structures *in vitro* to adequately mimic the *in vivo* niche. Specifically for neural applications, human brain cortical organoids (COs) require highly stratified neurons and glial cells to generate synaptic functions, and to date, most efforts achieve only fetal functionality at best. Moreover, COs are usually avascular, inducing the development of necrotic cores, which can limit growth, development, and maturation. Recent efforts have attempted to vascularize cortical and other organoid types. In this review, we will outline the components of a fully vascularized CO as they relate to neocortical development *in vivo*. These components address challenges in recapitulating neurovascular tissue patterning, biomechanical properties, and functionality with the goal of mirroring the quality of organoid vascularization only achieved with an *in vivo* host. We will provide a comprehensive summary of the current progress made in each one of these categories, highlighting advances in vascularization technologies and areas still under investigation.

## 1 Introduction

**FIGURE 1:**
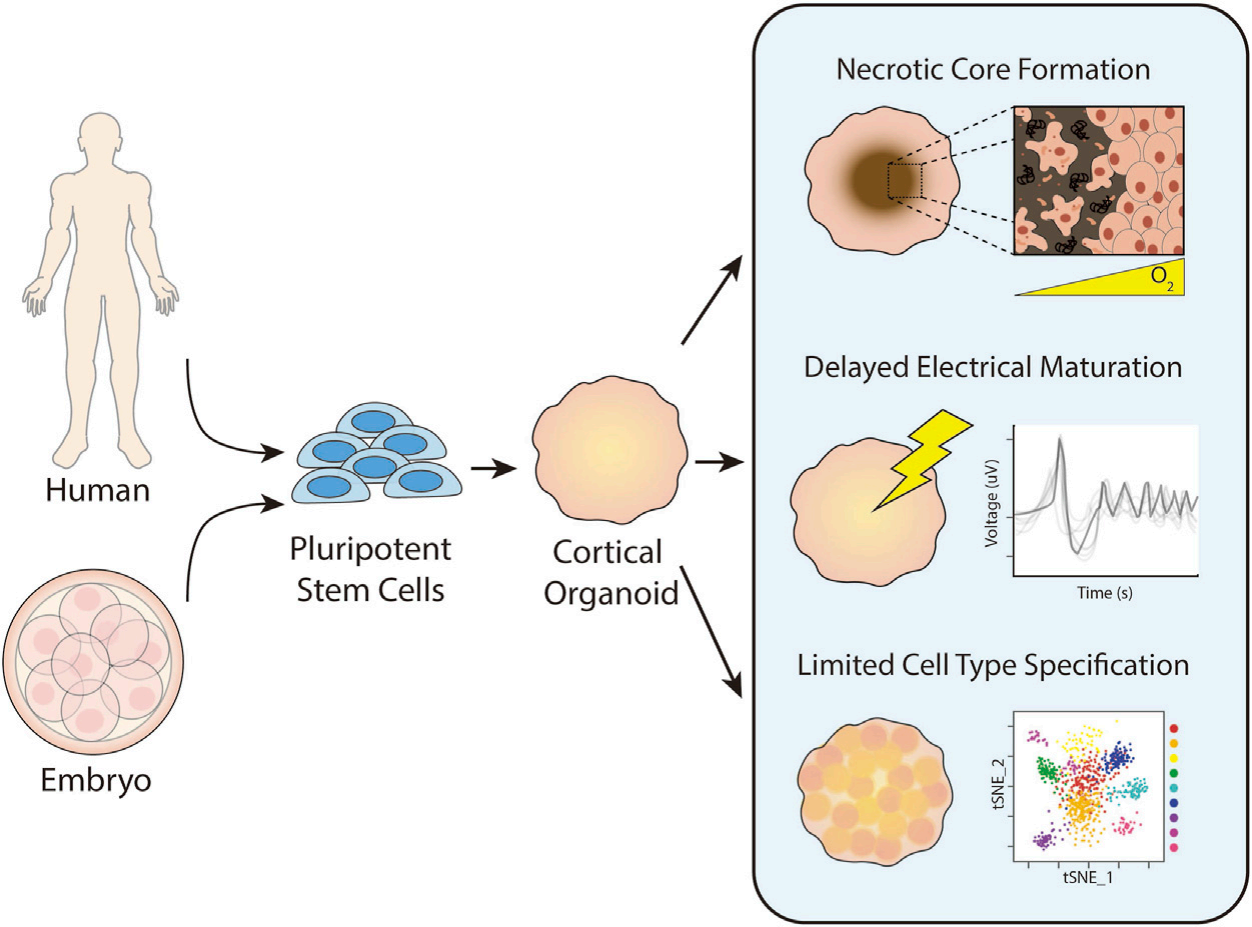
Historic challenges in cortical organoid culture. Cortical organoids are generated from pluripotent stem cells harvested from an embryo or reprogrammed from somatic cells. Upon prolonged culture, cortical organoids commonly form necrotic cores due to insufficient distribution of oxygen and nutrients. They also exhibit delayed electrical maturation with spontaneous action potentials occurring only after months of culture with minimal improvement over time. Finally, cortical organoids have limited cell type specification and differentiation after prolonged culture.

Organoids are self-organizing, three-dimensional cell clusters that are often derived from pluripotent stem cells (PSCs) (Rossi et al., 2018). They form with minimal direction from exogenous biochemical factors and create a tissue-specific niche where cells replicate some aspects of organ function or complexity (Qian et al., 2019). Embryonic and induced pluripotent stem cell (iPSC)-derived organoids provide a rare window into early cellular events that give rise to tissues and diseases (Lancaster and Knoblich, 2014; Pasca et al., 2015). Because organoids are generated from PSCs, strategies to prevent uncontrolled tumorigenicity, such as confirming the absence of pluripotent markers in differentiated cells or incorporating a suicidal gene to eliminate residual pluripotent cells, must be employed. Additionally, brain organoid protocols are still quite variable and thus, strong quality controls are necessary to reduce the experimental heterogeneity. Nevertheless, brain organoids have proven particularly useful for studying the human brain, which we have limited opportunities to observe and differs from those of animal models (Marshall and Mason, 2019). The cerebral cortex is perhaps the most interesting region of the human brain, as it is significantly larger and more complex than that of our evolutionary predecessors and regulates higher order functions like consciousness and thought. Cortical organoids (COs) have been used to study human-relevant neurodevelopment (Trujillo and Muotri, 2018), disease pathophysiology (Di Lullo and Kriegstein, 2017), and brain evolution (Giandomenico and Lancaster, 2017). Despite these advances, the absence of vasculature in organoid models leads to numerous problems beyond a lack of tissue complexity. A primary issue faced with organoids is the formation of a necrotic core due to insufficient mass transfer of oxygen and nutrients. Most cells can only survive ∼200 µm away from a vessel (Jain et al., 2005), but organoids can grow up to a few millimeters in diameter, resulting in heightened expression of hypoxia- and apoptosis-related genes and activation of metabolic stress pathways that negatively impact neural development and migration (Qian et al., 2019; Bhaduri et al., 2020). COs are also limited to immature fetal states, defined in part by less distinct cell types, rudimentary cortical layer formation, and variable electrophysiological activity (Luo et al., 2016; Qian et al., 2019; Puppo and Muotri, 2022) (Figure 1). Vascularizing brain organoids has alleviated these issues to varying degrees, but there remains a need for a reliable and effective method of CO vascularization.

**FIGURE 2:**
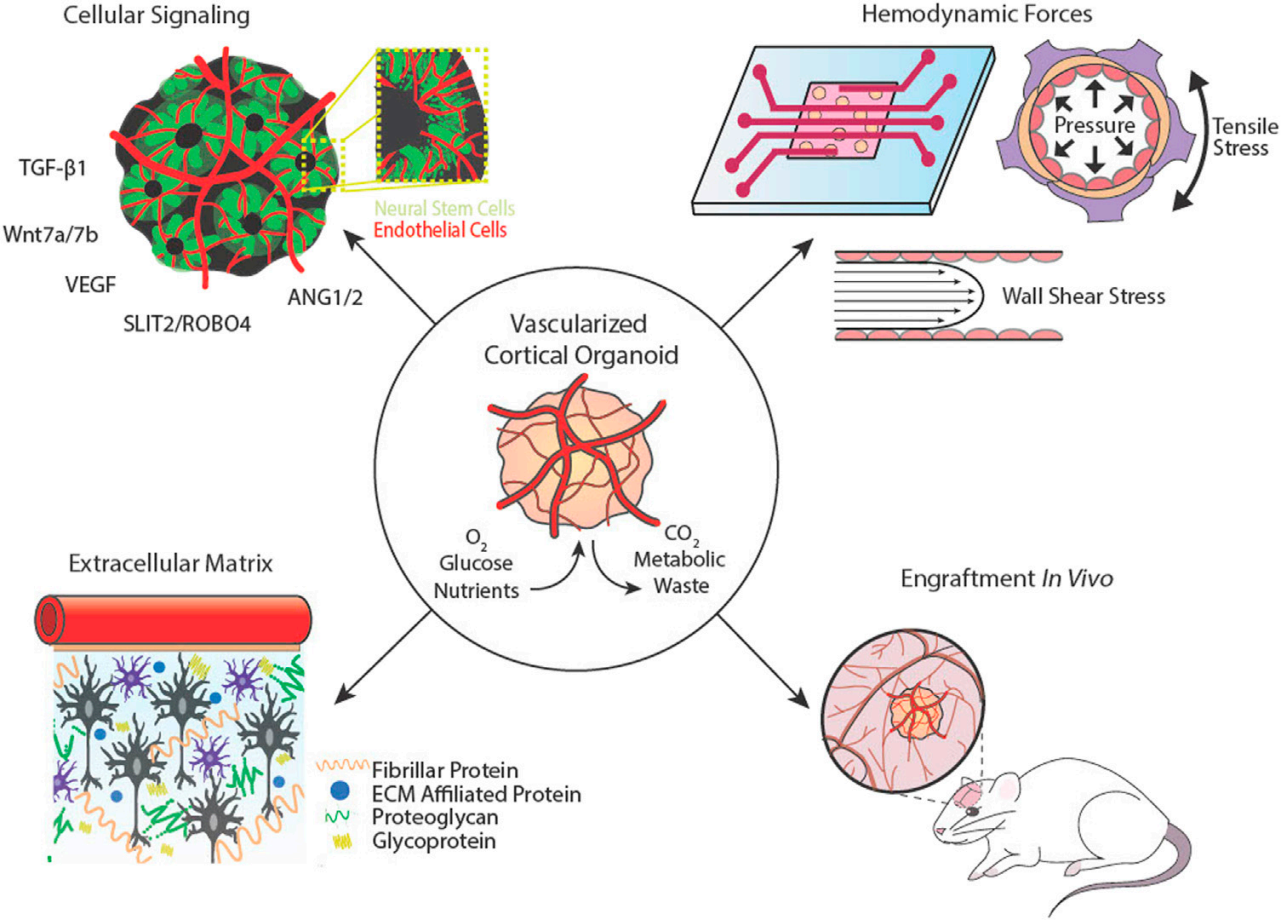
Cortical organoid vascularization components and considerations. Cellular signalling between neural and vascular cells is essential for complex network architecture. Hemodynamic forces that exude pressure and shear stresses on vessel walls can be mimicked using microfluidics. The extracellular matrix provides support and aids in mechanotransduction. Cortical organoids may be implanted into animal hosts to connect with or be invaded by host vasculature.

To successfully vascularize COs, it is important to consider both form and function. Vasculature primarily transports oxygen and nutrients essential for cellular survival and function, but also it plays roles in cell signaling, inter-organ crosstalk, and tissue-specific maturation (Carmeliet, 2005). The brain is one of the most densely vascularized tissues in the body, receiving 15–20% of the cardiac output to meet its high metabolic demands (Xing et al., 2017). Neurons, glial cells, and vascular cells work together to form a neurovascular unit, which regulates selective permeability of the blood brain barrier (BBB) and controls cerebral blood flow and stabilizes neuronal circuits via neurovascular coupling (Cipolla, 2009). The cerebral cortex exhibits unique vascular patterning in which large superficial vessels located in the pia mater branch off into venules and arterioles at 90-degree angles that penetrate the cortex columns. Small microvessels sprout between these penetrating vessels, forming dense microvascular beds located about ∼200 µm apart (Cipolla, 2009; Marín-Padilla and Knopman, 2011). This complex patterning is orchestrated by biochemical signals, cell-extracellular matrix (ECM) interactions, and biophysical cues provided by blood and cerebrospinal fluids (Figure 2). In this review, we examine the different approaches taken to vascularize COs, evaluate their successes and limitations, and identify key features that facilitate neurovascular development and can be leveraged to improve tissue models.

## 2 Biological self-organization of cortical vessels

**FIGURE 3:**
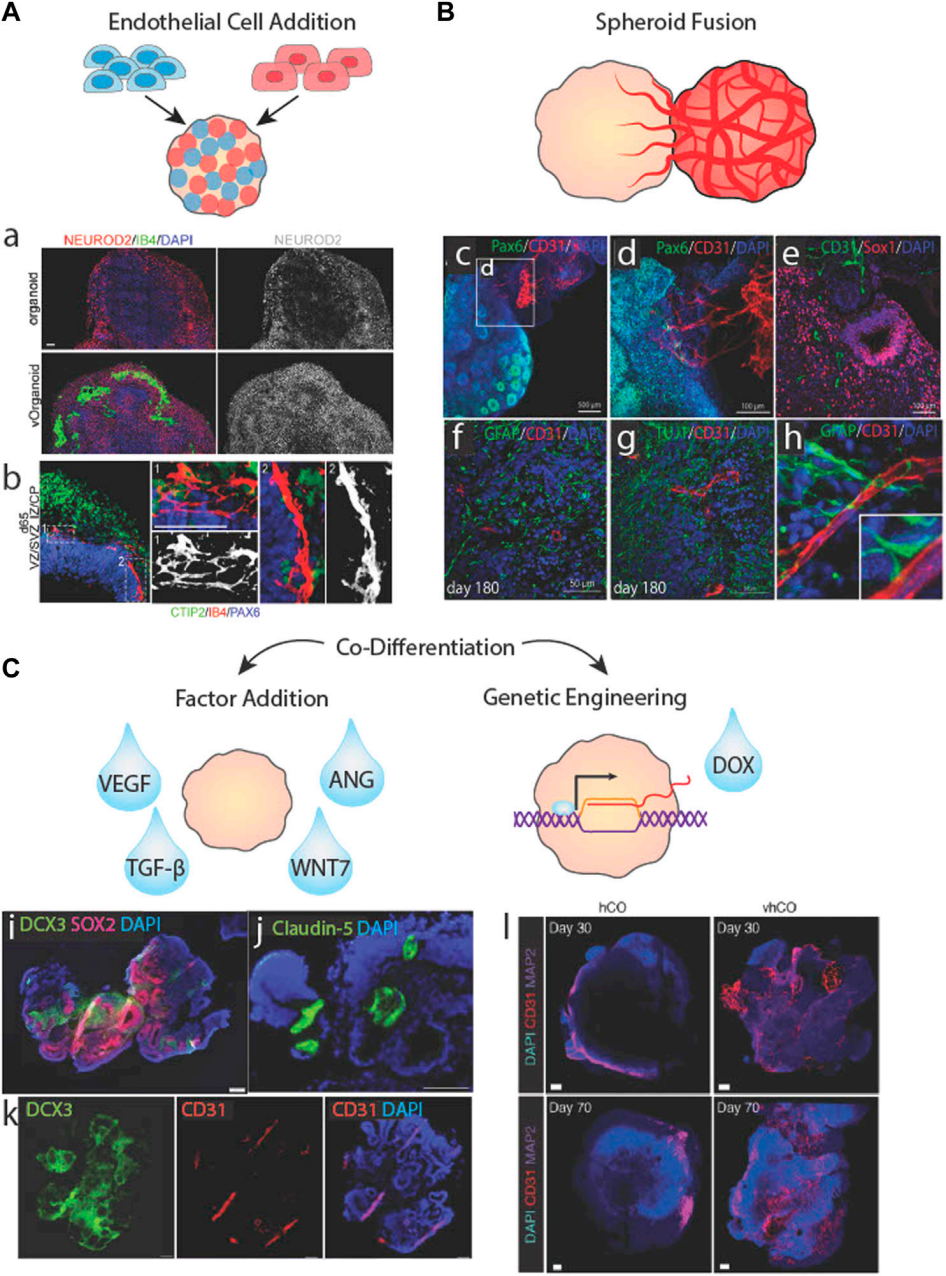
Biological approaches to brain organoid vascularization. **(A)** Schematic of organoid vascularization by mixing pluripotent stem cells with endothelail cells. a.b. Vascularized brain organoids generated by mixing HUVECs with stem cells showing localized clusters of vessel-like structures and vascular cells interacting with neurites. Adapted from (Shi et al., 2020). **(B)** Schematic of a brain organoid fused to a vascular spheroid. c.d.e. Vessels from a vascular spheroid invading the neuroepithelium of a brain organoid. f.g.h. Vessels from a vascular spheroid interacting with neurons and astrocytes/radial glia. Adapted from (Wörsdörfer et al., 2019). **(C)** Schematic of brain organoid vascularization by the addition of soluble factors or genetic engineering of inducible transcription factor systems. i.j.k Co-differentiated vascularized brain organoid stained for neural cell, vasucular cell, and tight junction markers. Adapted from Ham et al. (2020). Vascular markers in control (hCO) versus vascularized cortical organoids (vhCO), which carry an inducible Ets variant transcription factor 2 (*ETV2*) genetic system. Adapted from (Cakir et al., 2019).

The cellular and molecular signaling that direct cortical vasculogenesis *in vivo* are complex but offer a blueprint for the self-organization of vascular networks in COs (Figure 3). The goals of this process for COs are two-fold: First, most cell types of the BBB are neuroepithelium-derived, including astrocytes, pericytes, and neurons, and can arise spontaneously in COs. However, traditional COs lack the capacity to generate vascular endothelial cells (ECs) due to their mesodermal origins, so they must be added by other means. Second, signals must be provided to induce vascular network formation.

### 2.1 Vascular endothelial cell signaling in neocortical development

Proper brain development depends on the co-expansion of intercalated neural and vascular tissues. In neurodevelopment, ECs respond to pro- and anti-angiogenic factors to produce vascular networks and recruit other cells to the BBB. Vascular progenitor cells do not originate in the brain, rather, they are born in the vascular plexus that surrounds the central nervous system and ingress into the neural tube in response to hypoxic signals released by neural progenitor cells (NPCs). Subsequent signaling between vascular and NPCs activates neurogenesis and pro-angiogenic pathways that enable fetal brain growth and function (Carmeliet and Tessier-Lavigne, 2005; Ma et al., 2013).

In early corticogenesis, radial glial cells interact with ECs through both paracrine signaling and direct contact. Canonical Wnt signaling from radial glia regulates EC activation and vessel stabilization. In particular, Wnt7a and Wnt7b are expressed in a highly regulated and stage-specific manner that guides vascular network formation in the neural tube and forebrain (Ma et al., 2013). Radial glia also secrete transforming growth factor beta 1 (TGF-β1), an essential angiogenic factor that activates ECs, promotes EC migration, and supports tight junction formation (Siqueira et al., 2018). Simultaneously, Wnt family proteins, vascular endothelial growth factor (VEGF), and angiopoietins secreted from ECs regulate the self-renewal and differentiation of neural stem cells and induce the lateral expansion of the cortical epithelium (Carmeliet and Tessier-Lavigne, 2005). As a result of the signaling between radial glia and ECs, the regional density of radial glia correlates to vessel density and branching frequency (Ma et al., 2013). On the other hand, in late corticogenesis, neurons primarily coordinate vasculogenesis. VEGF, known as the master regulator of vascular growth, is released by both hypoxic tissues and neuronal axons (Carmeliet and Tessier-Lavigne, 2005). VEGF gradients activate ECs and induce the generation of tip cells, which delaminate the basement membrane and migrate *via* filipodia to make new vessel branches (Abe et al., 2019). VEGF-induced migration is inhibited by interactions between roundabout 4 (ROBO4) on EC membranes and SLIT2 on axons that subsequently upregulate cell adhesion and permeability pathways in ECs such as E-cadherin, β-catenin, and cadherin 5 (CDH5) and claudin 5 (CLDN5) (Bekes et al., 2017). But VEGF is not only important for ECs; it also plays key roles in neurogenesis and neuroprotection (Rosenstein et al., 2010). These are just a few of the numerous factors in the brain that guide both axon and vessel growth (Carmeliet and Tessier-Lavigne, 2005), though they illustrate the interdependence of axon and vessel signaling and how vessel and neuron density scale with each other (Tsai et al., 2009).

### 2.2 Addition of exogenous endothelial cells to cortical organoids

Terminally differentiated ECs in 3D environments spontaneously form tubular mesh networks in response to signals from neighboring ECs or other cells (Jeon et al., 2014), but it is important to consider what type of ECs are most appropriate to form networks that recapitulate BBB functions in COs. Human umbilical vein endothelial cells (HUVECs) are commonly used due to their commercial availability, ability to form tight junctions and tubes, and terminally differentiated state, negating the need for differentiation factors. However, HUVECs lack BBB-specific characteristics, including the expression of important surface and junction proteins and brain-specific transporters (Lee et al., 2020; Salmon et al., 2022). Some studies have noted that HUVECs in COs demonstrate transcriptional plasticity by adopting brain-specific vascular signatures, inducing the efflux transporter P-glycoprotein (Shi et al., 2020) and von Willebrand factor (vWF) (Kook et al., 2022). Skylar-Scott et al. (2022) also reported that HUVECs have difficulty adhering to iPSCs, possibly due to disparities in cell adhesion proteins, although high iPSC:HUVEC ratios in Shi et al. (2020) may have enabled the formation of hybrid spheroids (Figures 3Aa,b). The consequences of adding non-tissue-specific vascular cells to COs has not yet been explored, but with the previously described knowledge of cell signaling during neural differentiation, it is possible that adding such cells may disrupt neural patterning and differentiation. Primary human brain microvessel endothelial cells (BMECs) are another option but they have been minimally explored because they are difficult to acquire, quickly lose their brain-specific characteristics when cultured (Rubin et al., 1991), and form large, discontinuous vessels *in vitro* (Campisi et al., 2018; Lee et al., 2020). Similarly, BBB organoids formed from primary BMECs, astrocytes, and pericytes lack complex vascular morphology (Bergmann et al., 2018), likely due to the absence of tissue-specific biochemical and biomechanical signals provided by the brain microenvironment.

iPSC-derived ECs, on the other hand, are relatively readily available, can have the same genotype as their CO counterparts, and can undergo tissue-specific maturation, adopting brain-specific vascular signatures in the presence of neurons (Campisi et al., 2018; Salmon et al., 2022). iPSC-ECs transcriptomic identities and vascular functionalities depend on the ability of a protocol to reproduce the *in vivo* microenvironment while supplying sufficient differentiation cues (Pasut et al., 2021). Non-tissue-specific iPSC-ECs exhibit fundamental EC qualities, including EC-specific adhesion protein expression, solute impermeability, and tube formation both *in vitro* and *in vivo* (James et al., 2010; Patsch et al., 2015; Lee et al., 2020). However, they lack important qualities that contribute to the BBB and neurovascular development. Lippmann et al. (2012) developed a BMEC differentiation protocol that mimics the co-development of neural and vascular cells *in vivo* by combining soluble factors with physical cues from ECM substrates to co-differentiate neurons and ECs. The resulting iPSC-BMECs harbor highly organized tight junctions and elevated transendothelial electrical resistance. However, these cells neither express brain-specific transporters nor canonical EC genes and are unable to form continuous lumens *in vivo.* Notably, iPSC-BMECs have strong epithelial transcriptomic signatures, likely a result of naïve EC plasticity during co-differentiation with neuroepithelial cells, impairing proper EC-type specification (Lippmann et al., 2012; Lu et al., 2021). iPSC-BMECs can obtain canonical vascular EC characteristics *via* introduction of EC-specific ETS family factors, but this in turn erases their brain-specific qualities (Lu et al., 2021). Consequently, adding non-tissue-specific iPSC-ECs to COs and allowing them to acquire brain-specific characteristics *via* microenviromental signaling within the CO may be a very promising choice for CO vascularization.

Vascular network localization and density is impacted by the stage of CO differentiation when ECs are added and by their delivery method. ECs added during embryoid body formation are mixed as single cells with PSCs resulting in mixed spheroids (Figure 3A). Upon CO differentiation, the ECs assemble into vessels uniformly and densely across the organoid (Figures 3Aa,b,Cl), although these vessels may not be as interconnected or impermeable as some vascular networks generated using other methods (Cakir et al., 2019; Shi et al., 2020; Skylar-Scott et al., 2022). One concern of adding ECs during embryoid body formation is potential interference with neural induction, although Shi et al. (2020) conversely showed that such approaches may enhance neural differentiation. Still, some protocols opt to add vascular cells to COs after the neural induction phase by Matrigel encapsulation. This method relies on angiogenic and hypoxia chemokine gradients radiating from the CO core to guide vessel sprouting. However, minimal penetration into the CO has been reported, with the highest vessel density localized to the neuron-dense outermost layers (Pham et al., 2018). Minimal vessel penetration may be due to a number of causes: insufficient tip cell response, hypoxia signals, or NPC-EC signaling due to the distance between the ECs at the organoid surface and NPCs buried in the organoid body. A potential solution may be to incorporate biomaterials capable of releasing pro-angiogenic factors into the COs to encourage vessel sprouting, although this has not been attempted (Mastrullo et al., 2020; Seah et al., 2020).

### 2.3 Fusing vascular spheroids with cortical organoids

An alternative to mixed organoids is to propagate cell types separately as spheroids and subsequently fuse them together (Figure 3B). In this approach, vascular spheroids containing iPSC-ECs with fully formed networks have been fused to neural spheroids and cultured for long periods of time. Although the majority of neural and vascular cells remain regionally localized, a number of vessels sprout into radial glia-containing regions of the core after months of culturing (Figures 3Bc,e) (Wörsdörfer et al., 2019; Kook et al., 2022). Sprout penetration may be enhanced by EC-radial glia communication as a result of direct contact between the vascular cells and radial glial cells shortly upon fusion (Figures 3Bf–h). Despite relatively successful penetration and maturity of CO vessels formed *via* fusion, network formation and expansion was unresponsive to exogenous pro- and anti-angiogenic factors, (Wörsdörfer et al., 2019). As an alternative approach, Ahn et al. (2021) prevented the regional localization of vessels by dissociating HUVEC vascular spheroids prior to adding them to COs. Long vessels coated with pericytes were found throughout the organoid, but GFAP + astrocytes, although present, did not associate closely with these vessels. This may be due to the non-BBB characteristics of HUVECs or the lack of vessel branches and low vessel densities.

### 2.4 Co-differentiating endothelial cells and neurons

A third cell-based option found in the literature is to co-differentiate ECs and neurons within the same organoid (Figure 3C). This has been accomplished using exogenous differentiation factors, e.g., VEGF treatment, as early as 6 days after embryonic body formation to induce vascular differentiation without inhibiting neural differentiation and forebrain specification. However, this method does not produce a branched network, and only generates a few large, disconnected vessels that span the entirety of the CO (Figures 3Ci–k) (Ham et al., 2020). This result aligns with other studies that indicate VEGF is sufficient in generating vascular networks *in vitro* but composed of large and simple vessels. The addition of TGF-β1 or ANG1 significantly increased branching frequency and reduced vessel diameter (Jeon et al., 2014), although this has not been tested in vascularized CO culture.

An alternative to exogenous factors is to use genetically engineered PSCs harboring inducible transcription factor systems (Figure 3C). These cells undergo differentiation *via* lineage-specific gene expression when triggered by a chemical regulator like doxycycline. Ets variant transcription factor 2 (*ETV2*), a gene encoding a chromatin remodeling protein that activates tubulogenic pathways, has become a target for inducible-differentiation of vascular ECs (Palikuqi et al., 2020). Cakir et al. (2019) generated embryoid bodies containing unaltered iPSCs and iPSCs harboring *ETV2*-inducible expression systems then induced vascular expression *via* doxycycline at day 18 of CO differentiation. The result was a highly integrated, branched network spanning most of the organoid (Figure 3Cl). They also reported increased BBB marker expression and significantly improved electrophysiological activity. A reverse approach was taken by Dailamy et al. (2021) where iPSCs and inducible neurons were incorporated into vascular organoids and neuronal expression was induced on day 15 of vascular differentiation. This approach resulted in clusters of neural and vascular networks with some co-localization at their borders but without spanning the organoid uniformly. These biologically-based methods produced some vascularization, but as noted at the outset of this paper, a fully matured vascular network would contain small, highly branched vessels that span the entirety of the organoid. Since none of the methods resulted in such a network, other cues are likely important.

## 3 Extracellular matrix composition and mechanical properties

ECM-cell interactions also direct cortical vasculogenesis *in vivo*. The goal of ECM-based vascularization approaches is to recreate the brain’s extracellular microenvironment, which provides cells with structure to facilitate tissue growth and spatial-temporal regulation of biochemical and mechanical signals for patterning and maturation.

### 3.1 Extracellular matrix influence on angiogenesis during neocortical development

ECM is an essential component of any tissue microenvironment that supports differentiating cells. While it is perhaps an underappreciated element of the niche, brain ECM constitutes 20% of human brain mass (Rauch, 2007). The brain sees significant change in its ECM composition throughout development that affects mechanical characteristics such as diffusivity through extracellular spaces and tissue viscoelasticity, which regulates synapse development and functionality. ECM proteins help sequester cells and bind signaling molecules, in order to generate complex gradients and form the laminated structures of the cortex. In COs and the embryonic brain, the ECM maintains NPCs in regional layers and guides radial migration of neurons from the laminin-rich basement membrane of the cortical plate-like structure (Sood et al., 2016).

ECs secrete, degrade, deform, and are deformed by surrounding matrix to generate vascular networks in response to tissue needs (Du et al., 2016). ECs primarily produce and interact with fibrillar matrix proteins like fibronectin and collagen, which they reorganize to serve as scaffolding for migrating and elongating vessel sprouts (Zhou et al., 2008; McCoy et al., 2018). Despite its significant ECM mass, the brain has a relatively low concentration of fibrillar proteins compared to other tissues and the proportion of collagen to other ECM proteins decreases during development (Sood et al., 2016; Cho et al., 2021). In fact, fibrillar collagens in the adult brain almost exclusively surround the basal lamina of cerebral vessels. The bulk of brain ECM is composed of glycoproteins, which provide frameworks for synaptic junctions and play roles in regulating BBB permeability, and proteoglycans, namely chondroitin sulfates and heparan sulfate proteoglycans, which bind to hyaluronic acid molecules to generate soft aggregates that act as a supportive framework around cells (Maeda, 2015).

Beyond composition, the brain’s ECM exhibits unique structural and mechanical properties. The adult brain is an extremely soft, viscoelastic tissue, with an elastic modulus in the 100s of Pascal (Pa or N/m^2^; a unit of stiffness) (Fallenstein et al., 1969; Canovic et al., 2016). During development, the brain’s non-homogenous stiffness profile causes folding of the cerebral cortex (Budday and Steinmann, 2018), a characteristic important for cortical thickness and cellular density (Jalil Razavi et al., 2015). Similarly, organoids embedded in soft ECM hydrogels experience wrinkling and folding caused by differential swelling *via* solvent absorption. These features affect tissue growth, including organoid size, neuronal migration (Karzbrun et al., 2018) and number of rosettes (Cassel De Camps et al., 2022). Conversely, greater stiffness impairs overall growth and skews differentiation towards mature neuronal types (Cassel De Camps et al., 2022). While the effects of stiffness on EC growth in 3D tissues is understudied, it is well known that substrate stiffness greatly impacts ECs in 2D, with stiffer substrates inducing EC spreading and elongation, higher contractile forces, and increased proliferation (Yeung et al., 2005; Yeh et al., 2012). ECs are also sensitive to curvature, and will align and elongate along regions of high curvature (Cheng et al., 2019; Mandrycky et al., 2020). Ye et al. (2014) showed that HBMECs exhibit these effects more strongly than HUVECS.

### 3.2 Matrigel and extracellular matrix proteins

COs are commonly embedded in ECM proteins to provide structural support, promote neuroepithelial formation, and enhance growth and differentiation (Lancaster et al., 2013; Qian et al., 2016). While other matrix proteins have been used, Matrigel is the most common and, to our knowledge, the only matrix mixture currently used to vascularize brain organoids. Matrigel is a complex mixture of thousands of peptides but is mainly composed of laminin, collagen IV, and entactin. Matrigel also has significantly higher proportions of glycoproteins compared to native brain ECM but lacks important brain-specific secreted factors and ECM regulators (Cho et al., 2021; Simsa et al., 2021). However, its stiffness is tunable depending on its concentration, with Young’s moduli from 5 to 300 Pa (Chaudhuri et al., 2014), which encompasses the range of relevant brain ECM stiffness (Borries et al., 2020; Cho et al., 2021; Simsa et al., 2021).

Since angiogenesis depends on integrin-mediated interactions between vascular ECs and fibrillar ECM proteins, Matrigel is highly supportive for vascular network formation. A thorough ECM proteomic and biomechanical analysis of COs cultured in the absence of Matrigel has yet to be performed, so it is uncertain if cells within COs produce enough fibrillar proteins to sustain vascular networks. On the other hand, embedding COs in Matrigel impairs endogenous expression of ECM proteins, which may negatively impact neural patterning and maturation (Luo et al., 2016). Curiously, when COs are encapsulated with EC-containing Matrigel, complex and dense vascular networks form within the Matrigel shell but struggle to penetrate the CO (Pham et al., 2018), suggesting the CO microenvironment may not be favorable for angiogenesis.

Other ECM protein cocktails have not been used to vascularize COs, perhaps because they are less successful at facilitating CO differentiation. Strobel et al. (2021) showed that the addition of collagen to vascularized mesenchymal organoids did not enhance angiogenesis, and like Matrigel-coated organoids, organoids embedded in collagen secreted less ECM proteins. Similarly, Lancaster et al. (2017) showed that the addition of laminin, collagen, and entactin to Matrigel enhanced laminin-rich basement membrane maintenance and denser cortical plate organization similar to Matrigel alone, but using these matrix proteins in the absence of Matrigel did not recapitulate these effects. Even dissolving Matrigel into the organoid media has been shown to condense neurons and enhance radial organization (Lancaster et al., 2013), prolong NPC maintenance (Kadoshima et al., 2013), and reduce apoptosis (Qian et al., 2016). The exact reasons as to why Matrigel is particularly beneficial for CO culture are not well understood and Matrigel formulas are not fully defined, contributing to heterogeneity in brain organoids and making identification of prolific factors a challenge (Sood et al., 2016). Despite the unclear relationship between COs and Matrigel, ECs are capable of forming tubular networks on a variety of different purified ECMs, including collagen, laminin, and fibronectin (Andrée et al., 2019).

### 3.3 Decellularized extracellular matrices

Vascular network formation and stabilization has been improved in other organoid types by the addition of peptides from native tissue ECM, but this has not been tested in COs (Jin et al., 2022). Native tissue ECM hydrogels are thought to contain biochemical and biomechanical signals that recreate biological niches better than non-tissue-specific matrices (Cho et al., 2021). Cells cultured with tissue-matched ECM proteins maintain greater proliferation, phenotypic maintenance, and differentiation capacities than those grown on non-specific ECMs, highlighting the importance of ECM on cell specification and function (Zhang et al., 2009). Neurons cultured on brain ECM-containing substrates exhibit improved neural network formation and enhanced spontaneous firing. These effects are particularly exaggerated on substrates containing fetal brain ECM (Simsa et al., 2021), which has higher collagen content contributing to a stiffer mechanical profile than adult brain ECM (Sood et al., 2016). Like Matrigel, extracted brain ECM is significantly higher in collagen and other glycoproteins than native brain tissues, resulting in similar viscoelastic properties including low stiffness and high storage modulus (Borries et al., 2020; Cho et al., 2021; Simsa et al., 2021). Brain ECM-embedded COs have increased NPC populations, greater survival, stronger forebrain identity, and enhanced electrophysical maturity relative to Matrigel-embedded COs (Cho et al., 2021). In contrast, another study found that brain ECM-embedding causes differences in neuroepithelium budding but is equally as supportive of neuronal maturation as Matrigel (Simsa et al., 2021). Due to the sensitivity of ECs to ECM cues (Katt et al., 2018; Gifre-Renom et al., 2022), growing ECs on brain ECM may drive them towards BMEC specification or make them more compatible for vascularizing COs. However, significant further study is clearly needed to understand to what extent and how exogenous matrix modulates cell behavior within COs.

## 4 Fluid forces in perfusable vessels

Fluid forces give rise to, mature, and maintain vasculature in the cerebral cortex. Neural tissues receive nutrients and oxygen from the vasculature and expel wastes through cerebrospinal fluid in perivascular spaces. In static culture, solutes travel through organoids *via* diffusion, resulting in a lower concentration of nutrients like glucose, which is crucial for neural function, and oxygen, which has a diffusion limit of ∼200 µm (Magliaro et al., 2019), at their cores (Cho et al., 2021). Connecting COs to a flowing media source as a method of delivering nutrients and removing wastes would maintain a more stable metabolic profile, perhaps alleviating culture-related stress and enhancing maturation. Here, we will discuss the benefits of incorporating fluid flow into CO culture as well as current efforts to direct vasculogenesis in COs *via* perfusion.

### 4.1 Hemodynamic forces during neocortical angiogenesis

ECs lining blood vessel lumens are constantly exposed to fluid shear stresses and plasma interstitial forces resulting from blood flow. Astrocytes mediate the transport of interstitial fluids across the BBB into the ECM, exposing non-vascular cells to bulk convection flow carrying soluble nutrients. This highly regulated process depends on ECs modulating their functions in response to mechanical signals from hemodynamic forces. Shear stresses can alter EC cytoskeletal organization, gene expression, alignment, and migration. High shear stresses reduce EC proliferation and migration needed for early vessel formation, induce cell elongation, and initiate junction formation associated with vessel maturation (Dewey et al., 1981; Cucullo et al., 2013). These effects can be replicated *in vitro* by exposing ECs to shear stresses greater than ∼5 dyn/cm^2^ (Dewey et al., 1981). In the brain, shear stresses also regulate BBB-specific characteristics such as production of junction proteins that control permeability and transendothelial electrical resistance (Cucullo et al., 2013). The direction and magnitude of shear stresses direct microvascular network formation and autoregulation of cerebral blood flow. Interstitial flow can also contribute to angiogenesis as ECs guide newly forming sprouts towards regions of draining interstitial fluid flow in order to connect capillary beds (Galie et al., 2014). Interstitial flow *in vitro* increases vascular sprouting, network extension, and branching (Abe et al., 2019). Hence, the contribution that these forces have on vessel function cannot be ignored in attempting to form vessels in COs.

### 4.2 Convective flow in cortical organoid culture

Bioreactors incorporate patterns of fluid movement to improve the efficiency of nutrient transfer by convection rather than diffusion alone. Orbital shakers and spinning bioreactors are commonplace in labs now for organoid culture. In addition to reducing cellular stress, minimizing organoid variability, and supporting the growth of larger and more complex COs, they can support hundreds to thousands of organoids at a time (Qian et al., 2016; Monzel et al., 2017; Trujillo et al., 2019). However, these bioreactors lack the ability to balance velocities and shear stresses resulting in turbulent flow profiles, which are known to impact brain organoid development and morphology (Goto-Silva et al., 2019; Suong et al., 2021) and angiogenesis. Thus, microfluidic chips have been designed to produce specific and highly accurate flow profiles. When connected to peristaltic pumps, they can produce pulsatile, laminar flow with stable fluid shear stresses like those seen in blood vessels. Studies that have applied laminar flow to COs saw increased metabolic and dopaminergic maturation (Berger et al., 2018), organoid size, and electrophysiological activity (Cho et al., 2021) compared to COs grown in conventional bioreactors. In a study by Lee et al. (2020) laminar flow even induced the spontaneous formation of superficial but patent vascular networks on organoid surfaces. The next section will discuss how microfluidics have been used to generate perfusing vessels in COs.

### 4.3 Cortical organoids with vascular beds and tissue engineered vessels

**FIGURE 4:**
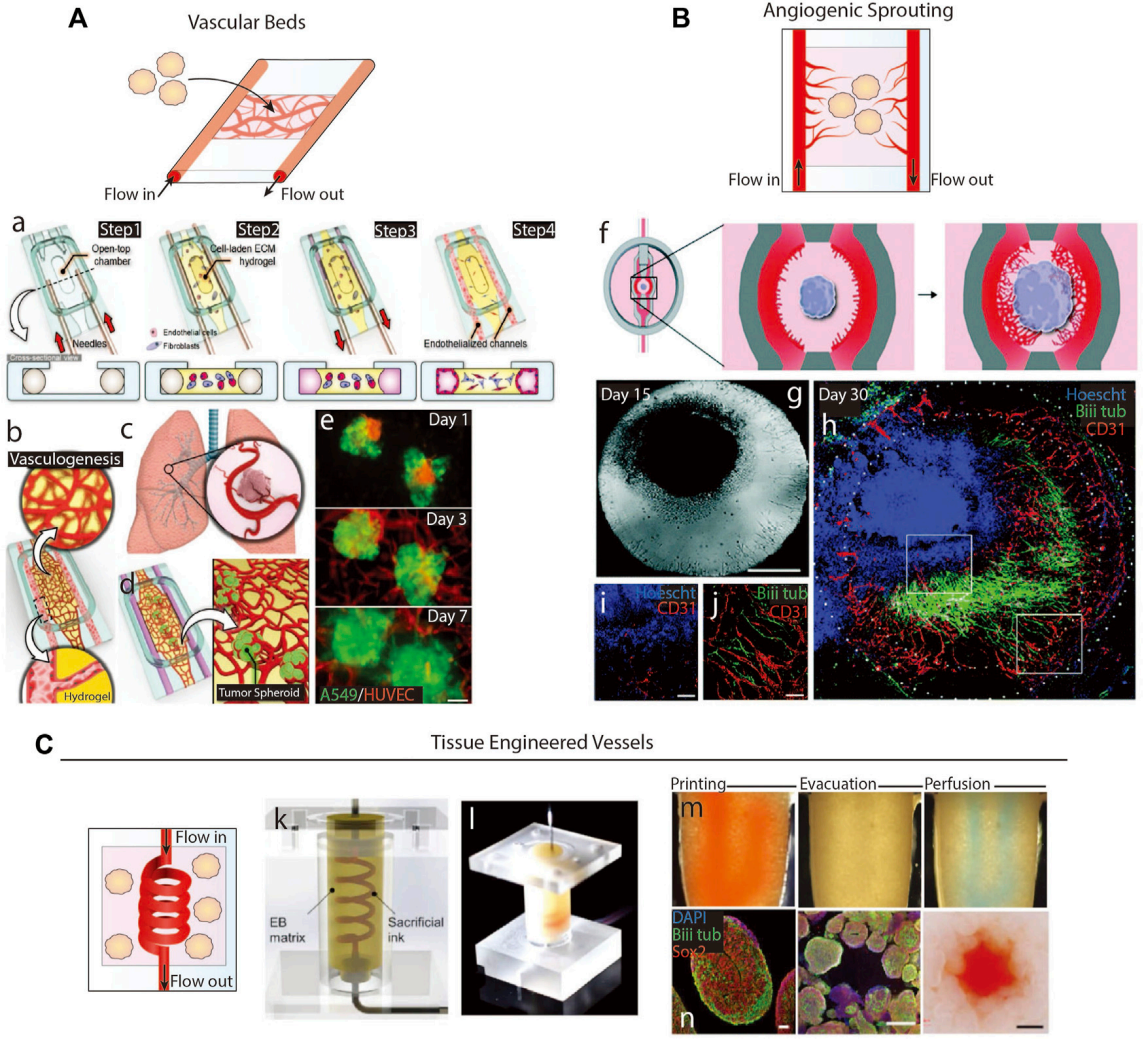
Microfluidics-based methods of vascularization. **(A)** Schematic of organoid vascularization using a microfluidic-generated vascular bed. a.b. Schematic showing the fabrication of a vascular bed. c.d.e. Introduction of lung tumor spheroids to the vascular bed. Adapted from (Paek et al., 2019). **(B)** Schematic of organoid vascularization by angiogenic sprouting towards a central brain organoid. g. Image of the organoid and angiogenic sprouts. h.i.j. Organoid and angiogenic sprouts fluorescently stained to show interaction between vessels and neurites. Adapted from (Salmon et al., 2022). **(C)** Schematic of tissue engineered vessel through a matrix containing organoids. k.l. A microfluidic device formed from a sacrificial ink in an embryoid body matrix. When a sacrificial ink is evacuated, the system is then perfusable. n. Images of brain organoids in the device after perfusion. Adapted from (Skylar-Scott et al., 2019).

One avenue of vascularizing organoids uses microfluidic devices to generate vascular beds through an organoid-containing matrix (Figure 4A). Shear stress is applied to two parallel EC-coated channels to induce EC migration into the central organoid-containing matrix until ECs from opposite channels meet to form a vessel. These systems have been applied to a variety of 3D tissues, including cancer spheroids (Figures 4Aa–e) (Paek et al., 2019), lung cell spheroids (Nashimoto et al., 2017), and COs (Salmon et al., 2022), and generate vascular networks with small inner-diameters and continuous lumens capable of transporting solutes (Paek et al., 2019; Lee et al., 2020). Similarly, organoids may be placed in an avascular hydrogel matrix between perfusing microfluidic channels from which ECs sprout towards the organoids in response to signaling gradients (Figure 4B). Salmon et al. (2022) showed that applying this method to COs resulted in short, highly branched vessels with heightened expression of functional vascular markers. While vessel invasion along COs' edges was frequent, less than half of the COs saw vessel invasion into their cores, suggesting that additional approaches are necessary to achieve fully integrated vessels (Figures 4Bf–h).

A second microfluidic method is to encapsulate organoids and a sacrificial scaffold in a hydrogel, remove the sacrificial scaffold, and perfuse media through the empty space (Figure 4C). These channels can be seeded with vascular cells, either by coating the sacrificial scaffold with cells or backfilling the empty space with cells, to generate a cell layer representing vascular luminal layers. Simple sacrificial scaffolds include needles, strings, or solid structures that can be manually removed from hydrogels, resulting in linear channels with diameters matching the structure’s diameter, usually greater than 100 µm (Neumann et al., 2003; Chrobak et al., 2006). Biomaterials can be used to make sacrificial scaffolds of more complex geometries and sizes by various fabrication methods. Common sacrificial materials include natural polymers, e.g., alginate and gelatin, or synthetic polymers, e.g., pluronics, Gel-Methacrylate, poly (vinyl alcohol), and poly (ethylene glycol), that undergo a phase transition when exposed to a specific environmental condition. For example, Skylar-Scott et al. (2019) 3D printed a gelatin scaffold through a matrix containing brain organoids, degraded the scaffold *via* heat, and backfilled the empty space with HUVECs (Figures 4Ck–n). Over 20 h of perfusion, no angiogenic sprouting into the organoid matrix was noted, but they found organoid viability increased. Similar studies using tumor fragments (Hu et al., 2018) and spheroids (Kwak and Lee, 2020) embedded in fibrin gels also saw increased viability, but only the later study observed sprouting. Even though Kwak & Lee only perfused their systems for 5 days, they used higher shear stresses at 5 dyn/cm^2^, which is sufficient to induce sprouting (Dewey et al., 1981). Vascular network formation was enhanced by coating the tumor spheroids with ECs, which sprouted towards the tissue engineered (TE) vessel in return. Alternatively, sprouting from the perfusing TE vessels may be achievable by inserting artificial chemokine gradients (Seah et al., 2020), since organoids may not produce strong enough signals to induce angiogenesis in low shear stress conditions.

### 4.4 Cortical organoid engraftment into non-human hosts

**FIGURE 5:**
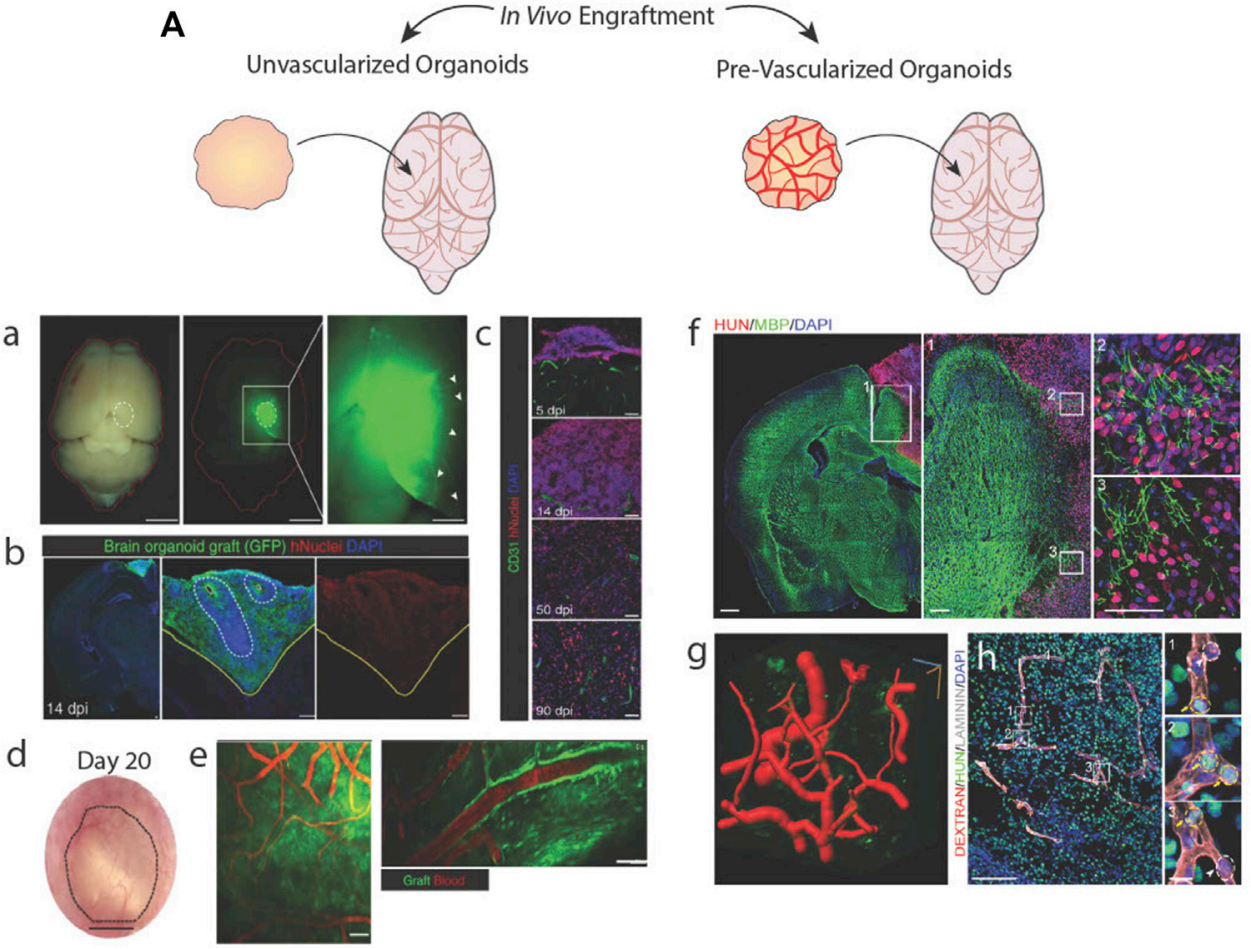
Brain organoid vascularization by engraftment into animal hosts. **(A)** Schematic of unvascularized or pre-vascularized organoids being engrafted in living mice brains. a.b. An unvascularized GFP + brain organoid located in a mouse brain stains positive for a human-specific nuclear marker. c.d. Engrafted brain organoid exhibits invasive host vessels. e. Vessels located in the graft perfuse blood. Adapted from (Mansour et al., 2018). A vascularized brain organoid located in a mouse brain stains positive for a human-specific marker and interacts with host tissues. g. Computational rendering of vessels within the organoid graft. h. Vessels within the graft perfuse a labelled dextran injected into the host mouse. Adapted from (Shi et al., 2020).

Organoids implanted into animal hosts offer a less controlled but efficient method to becoming vascularized. A host’s vessels invade an engrafted organoid in response to proangiogenic signals, in order to prevent graft death (Figure 5A). Mansour et al. (2018) transplanted brain organoids into mice cortices and identified vascular invasion as early as 5 days post-transplantation that directly correlated with decreased apoptosis and survivability of the graft (Figures 5Aa–e). Engrafted organoids also were invaded by host immune cells and made synaptic connections with host neurons across different regions of the brain, resulting in neuronal synchronization. Extensive host vascularization also increases neuronal differentiation and activity, enhanced survival (Daviaud et al., 2018; Bao et al., 2021), and reduced cellular stress (Bhaduri et al., 2020).

Engraftment of pre-vascularized tissues supports better vessel invasion and graft survivability than avascular grafts (Figures 5Af–h) (Ben-Shaul et al., 2019; Shi et al., 2020; Wassmer et al., 2022). With brain organoids, such experiments have been performed in mouse cortical tissue (Pham et al., 2018; Cakir et al., 2019; Shi et al., 2020), subcutaneous tissue (Cakir et al., 2019), and chicken chorion allantois membranes (Wörsdörfer et al., 2019) to highlight the ability of *in vitro*-derived vessels to sustain hemodynamic mechanical forces and retain solutes as evidence functional luminal junctions. However, it is uncertain if *in vitro*-derived vessels obtain these capabilities because of *in vivo* hemodynamic forces or host-graft cell signaling rather than acquiring these mature functional characteristics as a result of *in vitro* vascularization efforts.

## 5 Successes and challenges in cortical organoid vascularization

To assess the accomplishments and describe future directions that CO vascularization should take, here we summarize what we believe will be necessary to achieve fully functional CO vascularization in the context of current methods (Table 1). These criteria cover vascular architecture, mature BBB characteristics, and neurovascular function. Of these categories, engrafted organoids *in vivo* are most successful. They vascularized robustly and show strongest evidence of vessel maturity and perfusability, but because they rely on non-human hosts, COs vascularized *in vivo* are rendered imperfect representations of human neurovasculature and unsuitable for modeling human disease or development. For this reason, they are not a reliable approach to CO vascularization, although they may provide insight on biological cues needed to further the vascularization field. We will continue with a comparison of *in vitro* methods.

**TABLE 1:** Comparison of organoid vascularization methods. Table outlining the success of each organoid vascularization method at achieving realistic vessel architecture, perfusability, and mature blood brain barrier characteristics.

	Biological self-organization			Microfluidic engineering			Living tissues
	Endothelial Cell Addition	Spheroid Fusion	Co-Differentiation	Vascular Beds	Angiogenic Sprouting	Tissue Engineered Blood Vessels	*In Vivo* Engraftment
Human-Only	Yes	Yes	Yes	Yes	Yes	Yes	No
Integrated	Variable	Variable	Variable	Variable	Variable	No	Yes
Complex	Yes	Yes	Variable	Yes	Yes	No	Yes
Continuous	Yes	Yes	Variable	Yes	Yes	Yes	Yes
Perfusable	No	No	No	Yes	Yes	Yes	Yes
Tight Junction Formation	Yes	Yes	Yes	Yes	Yes	Yes	To be determined
Reduced Hypoxia	Variable	To be determined	Variable	Yes	Yes	Yes	To be determined
Reduced Necrosis/Apoptosis	Variable	Variable	Variable	To be determined	To be determined	Yes	Yes
Accelerated Neural Maturation	Variable	Variable	Variable	To be determined	Yes	To be determined	To be determined
Pericyte Recruitment	Variable	Yes	Variable	To be determined	To be determined	To be determined	To be determined
Enriched Astrocyte Population	Variable	Yes	Variable	To be determined	To be determined	To be determined	Yes
Enhanced Electrical Activity	Variable	To be determined	Variable	To be determined	To be determined	To be determined	Variable

Cortical microvasculature is dense, highly branched with narrow vessels and a continuous interconnected lumen. *In vitro* approaches using self-organizing vessels have reached the greatest success to date in recapitulating these features. The best of these being EC addition during organoid formation, which results in dense and highly branched networks that span the majority of the organoid, with vessels preferentially localized in outermost neuronal layers (Cakir et al., 2019; Shi et al., 2020). Yet, biologically-based self-organization approaches may be incapable of accomplishing the same level of vessel interconnectedness as models that incorporate flow, since the development of this feature relies on fluid forces. Methods that utilize angiogenic sprouting–vascular spheroid fusion, vascular bed devices, and angiogenic sprouting devices–benefit from incorporating 3D pre-assembled vascular networks, which arise from a process we do not yet fully understand and thus have been challenging to recreate in organoids. TE vessels currently fall short in producing realistic vasculature, often generating vessels adjacent to organoids rather than through them. This lack of integration is accompanied by a general lack of complexity, as TE vessels are usually significantly larger than cortical microvessels and lack branches due to limitations by their fabrication methods (Chen et al., 2021). Realistic vascular architecture may remain unachievable in the tissue engineering field until fabrication methods capable of smaller, more exact features are available.

Maturity of neurovascular tissue is indicated by neuronal electrical activity, cell-type specification, and properties of the BBB, such as EC tight junction formation and recruitment of pericytes and astrocytes. ECs in COs typically accelerate neural differentiation, decrease cell death, and enhance spontaneous electrical activity in COs (Cakir et al., 2019; Shi et al., 2020; Kook et al., 2022), regardless of vascular architecture. Most biological vascularization methods also note mature BBB characteristics, focusing on junction-associated protein expression and changes to astrocyte populations. Vessels formed *via* sprouting from a perfusion source, e.g., those that use microfluidic devices, presumably have fully formed tight junctions necessary for sustaining fluid flow, therefore this is not always reported. However, the effects of vascularization of COs by engineering methods as a whole are understudied. While convective flow is known to be highly beneficial for CO culture (Qian et al., 2016; Monzel et al., 2017; Trujillo et al., 2019; Cho et al., 2021), most microfluidics-based organoid vascularization methods focus on reducing hypoxia and neglect addressing other areas of neurovascular maturity (Skylar-Scott et al., 2019). Thus, without proper evaluation of maturity criteria in engineering-based approaches, we can only conclude that biological self-organized vessels currently have the greatest benefits towards neurovascular maturity.

Since sustaining blood flow is a key function of vasculature, it is important for vascularized COs to be patent and perfusable. COs vascularized by biological approaches are unable to be connected directly to a flow source or fluidics pump. Some studies add fluorophore-conjugated dextrans to culture media and allow these sugars to flow into biologically-vascularized COs *via* diffusion or convective flow in bioreactors, suggesting that the vessels are patent (Cakir et al., 2019; Shi et al., 2020). Vascularization methods dependent on microfluidic systems provide the most reliably patent and perfusable vasculature. Because they are hooked up to pumps with controllable perfusion rates, realistic shear stresses and flow profiles and a constant supply of nutrients are easily achievable. Thus, microfluidic-based methods are the best models for replicating hemodynamic forces and perfusion.

## 6 Conclusion

Vasculature provides tissues with means to transport nutrients and oxygen, metabolites, and cellular signals that facilitate growth and maintain homeostasis. Without vasculature, brain organoids are limited in their differentiation capabilities, morphological patterning, and electrophysiological functions. Here, we have identified different methods of CO vascularization from the most recent and notable published studies and evaluated their successes and drawbacks as they relate to cortical vasculature *in vivo*. Each of the discussed methods has their own advantages, but none fully capture the form and function of an *in vivo* neurovascular unit. Nevertheless, these studies have elucidated important relationships between cells and biochemical and biophysical forces and highlighted the capabilities of *in vitro* cultures in recapitulating aspects of the neurovascular microenvironment. Moving forward, the CO vascularization field may benefit from combining current approaches and looking into the use of new biomaterials and ECM technologies as well as improving existing techniques.

## Data Availability

The original contributions presented in the study are included in the article/Supplementary Material, further inquiries can be directed to the corresponding authors.
